# Role of Executive Function in Response to a Problem Solving Based Psychoeducational Intervention in Adolescents with Psychosis: The PIENSA Trial Revisited

**DOI:** 10.3390/jcm8122108

**Published:** 2019-12-02

**Authors:** Marta Rapado-Castro, Carmen Moreno, Ana Ruíz-Sancho, Francisco Camino, Celso Arango, Maria Mayoral

**Affiliations:** 1 Department of Child and Adolescent Psychiatry, Institute of Psychiatry and Mental Health, Hospital General Universitario Gregorio Marañón, School of Medicine, Universidad Complutense, IiSGM, CIBERSAM, 28009 Madrid, Spain; cmoreno@hggm.es (C.M.); carango@hggm.es (C.A.); maria.mayoral@iisgm.com (M.M.); 2 Melbourne Neuropsychiatry Centre, Department of Psychiatry, University of Melbourne & Melbourne Health, Victoria 3053, Australia; 3 InterAcción—Psychotherapy, Groups and Personal Development Counselling and Psychotherapy Centre, 28009 Madrid, Spain; anamarusa@gmail.com; 4 VocAcción—Group Processes and Institutional Consulting Counselling and Psychotherapy Centre, 28009 Madrid, Spain; 5 Physiology Department, Faculty of Biological Sciences, Universidad Complutense, 28040 Madrid, Spain; francisco.camino90@gmail.com

**Keywords:** psychosis, adolescents, PIENSA program, executive function, psychoeducation, problem-solving, cognition

## Abstract

An improvement in negative symptoms and a reduction in the number of visits to the emergency department have been reported in a problem solving based psychoeducational group intervention (PE) for adolescents with psychosis relative to a nonstructured group (NS). One of the factors that may play a role on the response to PE treatment is executive function (EF), a crucial cognitive domain for problem-solving performance. We aimed to examine the role of EF in response to PE treatment versus an NS group. We examined the associations between changes in cognition and in clinical/functional variables within each treatment group using Spearman-ranked and partial correlation analyses. A total of 22 individuals (mean age: 16.3) were randomized to PE (*N* = 10) and NS (*N* = 12). We found an association between improvements in EF performance and a reduction in positive symptoms (r_s_ = –0.756, *p* = 0.030 for semantic fluency), reduction in negative symptoms (r = 0.758, *p* = 0.029 for semantic; r_s_ = –0,733, *p* = 0.025 for verbal fluency), and reduction in the number of visits to the emergency department (r = –0,743, *p* = 0.035 for semantic fluency) in the PE group. No associations were found in the NS group. Our results suggest that EF may play a role in the specific improvements observed in the PE group. This may have implications in the development of new areas of clinical intervention focusing on the role of cognitive functioning in response to psychosocial treatments in psychosis.

## 1. Introduction

Continued beneficial effects in clinical and functional outcomes have previously been reported for participants in a nine-month problem solving based psychoeducational group intervention (PE) for adolescents with psychosis and their families (i.e., the PIENSA program [1]), at the end [2] and two years after the intervention [3], relative to a nonstructured group intervention (NS). We found an improvement in negative symptoms and a reduction in the number of visits to the emergency department in the group of adolescents with psychosis who participated in psychoeducational (PE) treatment compared to the nonstructured (NS) group at the end of the intervention [2]. Participants in the PE group also had a decreased number of visits to the emergency department at two-year follow-up relative to participants in the NS group [3]. 

One of the factors that may play a role in the response to PE treatment is executive functioning performance. Executive function involves the orchestration of high-order cognitive processes that are necessary to deliberately solve a problem and/or to respond to the demands of daily life [4]. Deficits in these core processes have consistently been shown in early psychosis [5,6,7]. Our own previous studies in an independent group of adolescents with psychosis have shown that executive functioning performance is altered at the time of the first episode [8], and, although function in this cognitive domain improves at follow-up, the degree of the impairment relative to healthy controls remains stable over the first two years of the illness [9,10]. Specifically, studies on the first episode psychosis have supported the notion that executive functioning deficits are predictors of significant social, functional, and vocational disability leading to a poor quality of life in this population [11,12,13,14,15]. Moreover, executive functioning performance has been strongly associated with the severity of negative symptoms in these patients [16,17], which has an impact on interpersonal behavior, work, household functioning, and community development [18,19,20]. In particular, studies have found consistent, significant cross-sectional associations between executive functioning performance and measures of social problem solving and daily life activities in adults with schizophrenia. Poorer cognitive flexibility and verbal fluency abilities are associated with worse daily life problem solving and independent living skills in schizophrenia [21,22,23]. Specifically, deficits in attention, reasoning, cognitive flexibility, response inhibition, and verbal fluency and processing speed can limit an individual´s ability to lead a normal life [24]. Executive impairments can interfere with the ability to understand and internalize the information and skills trained in specific psychotherapies for psychosis and, thus, prevent applying them in everyday life [25].

Problem-solving training strategies are a core part of cognitive remediation approaches for patients with psychosis. They have traditionally included in these programs to enhance the patient’s skills to cope and manage their daily life dilemmas. The aim is to assist participants to adapt to novel situations, orientating and redirecting their behavior in order to set and properly archive their goals by generating, evaluating, and choosing between alternative courses of action [26,27]. The inclusion of this key component in cognitive remediation programs has demonstrated greater positive effects than those approaches only based on “drill and practice” [28]. Particularly, the implementation of a problem-solving framework within cognitive remediation programs has shown not only to improve executive deficits but also to contribute to successful and sustained cognitive and functional recovery [18,29,30,31,32,33]. Moreover, the implementation of these strategies together with multifamily group psychoeducation has shown better results than cognitive remediation or psychoeducation alone in improving the course of psychosis [34] 

In this context, problem-solving strategies [35] have been defined as an essential component of our PE group intervention [1]. Although the inclusion of this component within the PIENSA (*Programa de Intervención en Psicosis Adolescente;* Intervention Program in Adolescents with Psychosis) clinical program was aimed to prevent relapses and facilitate self-management in stressful situations, plausible remediation of executive function through enhancement of problem-solving skills may play a role in the observed beneficial effects of the PE intervention [2,3]. Previous studies have explored the role of cognitive performance on functional variables such as rate of employment [36], vocational outcomes [37], functional recovery, and treatment satisfaction [38]. However, to the best of our knowledge, no previous studies have explored the role of executive functioning performance in the response to PE interventions in psychosis. The present study aims to examine the role of executive function in response to a problem solving based PE treatment relative to an NS group intervention in participants with psychosis who underwent baseline and follow-up cognitive assessments in the context of the PIENSA randomized controlled clinical trial (NCT02101372). We hypothesized that an improvement in executive functioning performance over time (i.e., an improvement in problem-solving skills) would be associated with beneficial effects (such as a reduction in symptoms and number of visits to the emergency department) observed in the problem solving based PE group.

## 2. Experimental Section

The main clinical trial has comprehensively been described elsewhere [1,2,3]. In brief, this was a two-year follow-up, randomized, rater-blinded, outpatient trial on the efficacy of a problem solving based PE group intervention implemented in parallel for patients with early-onset psychosis and their families compared to an NS group intervention. In this context, all cognitive, clinical, and functional ratings were rater-blinded. The method of randomization was random number generation by computer program. After providing written informed consent, all randomized participants received PE or NS group intervention for 9 months.

The authors assert that all procedures contributing to this work complied with the ethical standards of the relevant national and institutional committees on human experimentation and with the Helsinki Declaration of 1975, as revised in 2008.

Dr. Marta Rapado-Castro had full access to all study data and takes responsibility for the integrity of the data and the accuracy of the data analysis.

Clinical trial registration information: Intervention Module AGES (AGES-CM) (http://clinicaltrials.gov/); NCT02101372.

### 2.1. Study Participants

The original study was offered to 90 adolescent outpatients with early onset psychosis. A total of 55 patients were finally included in the main trial, allocated to PE (*N* = 27) and NS (*N* = 28) group interventions [2,3]. 

The inclusion criteria for patients were age between 14 and 18 years, living at home with either one or both parents, caregivers, or legal guardians, and the presence of at least 1 positive psychotic symptom (delusions or hallucinations) before 18 years, with diagnosis of a psychotic disorder according to the Diagnostic and Statistical Manual of Mental Disorders, fourth version, text revised DSM-IV-TR. The exclusion criteria were any neurological developmental disorder and inability to engage in conversation or read in Spanish that might interfere with the progress of group treatment. Drug abuse or dependence was also an exclusion criterion, but not drug use at the time of the intervention.

The current study examined the role of executive functioning performance in clinical outcome measures over time. Therefore, only those participants out of the original sample (*N* = 55) who completed both baseline and two-year follow-up neuropsychological assessments (*N* = 22) were included in the present study. Of those, 20 had a first episode of psychosis, and 2 had had previous episodes (1 hospitalization (*N* = 20); 2 hospitalizations (*N* = 2)). Participants who had valid baseline and longitudinal cognitive data (*N* = 22) had a higher Positive and Negative Syndrome Scale (PANSS) negative symptoms score at baseline (19.57 ± 7.34) than those who did not complete the corresponding cognitive assessments (15.00 ± 6.60; t = 2.34, *p* = 0.023). They did not differ significantly in terms of positive symptoms, functioning, antipsychotic medication, number of previous episodes, number of days hospitalized, age, sex, race, diagnosis, treatment group assignation, or number of visits to the emergency department at baseline (results not shown). 

### 2.2. Intervention Program in Adolescents with Psychosis, the PIENSA Program

Group interventions were implemented in parallel in 2 separate groups (for parents and for adolescents). PE intervention focused specifically on problem-solving strategies and structured psychosis-related information to manage daily life difficulties associated with the disorder. To mitigate crises and to prevent relapses, written material (i.e., a handbook of psychosis [39]) was provided. The NS intervention used a supportive group approach aimed to connect adolescents and parents facing similar challenges, thus enabling members to share experiences and advice. The same two therapists delivered both the PE and NS group interventions for patients, and two other therapists delivered both (i.e., PE and NS) to parent groups. A detailed description of the study design of the PIENSA clinical trial has been published elsewhere [1].

#### 2.2.1. Problem Solving Based Psychoeducational (PE) Group Intervention

The PE group consisted of a multifamily intervention including an initiation and a group phase. The initiation phase comprised three individual sessions of 50 min each that allowed for the exploration of current dilemmas and the establishment of therapeutic alliance. The group phase included 12 biweekly, 90 min structured sessions focused on the development of problem-solving strategies to manage daily life difficulties. These structured sessions included an informal chat in which all the group members could talk about their difficulties or concerns. Group leaders picked out an individual dilemma to be solved in the group, making use of the problem-solving strategies that were at the core of each session [1]. The use of problem-solving strategies aims to help participants to take a more active role in their lives by teaching them skills to effectively make decisions regarding their psychotic disorder (i.e., taking medication or managing its plausible side effects) and achieve their goals (i.e., completing or finalizing their studies). By exemplifying this problem-solving approach with a particular dilemma (i.e., “I do not know how to talk to others about my bipolar disorder”), participants may learn to apply/generalize it to any other dilemmas they may encounter (i.e., “I do not know how to say no to alcohol and drugs”). This may empower them to face difficulties more independently, increasing their confidence and agency to manage different aspects of their lives related to the disorder [40]. 

The core components of the problem-solving strategies [35] implemented in the PE group are described as follows [26,27,29]. (1) Clear definition of the dilemma. Participants, together with their group leader, identified a dilemma and the negative or ineffective answer that it generated in the targeted individual. In cognitive terms, the definition of a problem requires an adequate orientation of the attention capacity towards what is happening/worrying participants; an adequate abstraction capacity in order to represent the problem; planning, monitoring, and inhibition to inhibit nonrelevant information; and decision making to focus on defining the specific trouble that is happening with themselves. (2) Brainstorming. Once the problem had been clearly formulated, the group members were encouraged to generate as many potential solutions that came to their minds for the defined situation. Verbal fluency and processing speed are essential at this point in order to evoke the greatest number of possible solutions through verbal expression. (3) Evaluating solutions. Potential solutions generated in the previous step were evaluated through careful examination of their plausible pros and cons. This allows for the exercitation of attention and abstract thinking in order to represent and actually evaluate the adequacy of each solution. (4) Taking action. The PE group session ended with a clear definition of an action plan drawn up by the whole group based on the solution selected by the individual whose situation had been examined. This requires the activation of the so-called planning abilities. Then, the selected solution/ plan needed to be executed or carried out between sessions to see if it really worked for the target individual. (5) Task review. The target participant from the previous session was invited to review/evaluate the adequacy of the attempted solution. If it was not possible to implement or if it failed, then another solution from those reviewed in the previous session might be selected and implemented more effectively and discussed in the following session. In this phase, cognitive flexibility plays a fundamental role in helping participants to adapt to the changing demands of the environment. 

#### 2.2.2. Nonstructured Intervention 

The NS also included both an initiation and a group phase where facilitators did not follow a structured model or provided written material. Instead, we encouraged participants to talk about their personal experiences to help and support each other. The help could take the form of sharing coping strategies, establishing social networks, providing relevant information, and/or listening to other’s experiences. Thus, no specific tasks that may have an effect on cognitive functioning were conducted other than an informal talk in the context of a support NS group. 

Both the PE and NS group interventions complemented current individual psychiatric management and psychopharmacological treatment.

### 2.3. Clinical Assessment and Outcome Measures

Diagnosis was established at baseline according to DMS-IV-TR criteria using the Spanish version of the semistructured interview for children and parents, the Kiddie Schedule for Affective Disorders and Schizophrenia for School-Age Children—Present and Lifetime version (K-SADS PL) [41], and was confirmed using the same instrument at 2 years of follow-up when patients were younger than 16 years or a structured clinical interview for DSM-IV Axis I disorders (SCID-I) when patients were older than 16. Three main diagnostic categories were established for descriptive purposes only: schizophrenia spectrum psychosis (SSD; *N* = 10), affective psychosis (*N* = 9), and other types of psychoses (*N* = 9).

Symptom severity scores, global functioning and number of relapses in terms of the number and duration of hospitalizations and number of visits to the emergency department, were included as outcome variables in the present study. Severity of positive and negative symptoms was assessed using the Positive and Negative Syndrome Scale (PANSS) [42,43]. Global functioning was measured using the Children Global Assessment of Functioning (C-GAS) Scale [44]. Number and duration of hospitalizations and number of visits to the emergency department were collected through a custom-designed questionnaire and subsequently corroborated using medical records. These evaluations were blindly applied by experienced psychiatrists in child and adolescent psychiatry before the intervention (i.e., at baseline) and at two-year follow-up.

### 2.4. Neuropsychological Assessment

Each individual underwent a comprehensive neuropsychological assessment at baseline and at two years of follow-up, including those cognitive functions consistently described as being affected in psychosis [5,6] (i.e., attention, working memory, memory, and executive function). Participants completed this cognitive evaluation in the context of a parallel, longitudinal, adolescent, first-episode psychosis study aimed to examine clinical, neuropsychological, biological, and prognostic factors in these participants with psychosis [45]. Of those, only executive functioning measures were selected in order to measure the change in performance of this cognitive domain over time (two-year follow-up minus baseline). Specifically, the following cognitive tests and their corresponding derived neuropsychological variables were used: Trail Making Test (TMT) [46] derived score (i.e., TMT part B minus TMT part A; a relatively pure indicator of executive control [47,48]); Wisconsin Card Sorting Test (WCST; i.e., total number of errors, perseverative errors, and categories completed, including indicators of cognitive flexibility, executive control, planning, monitoring, and decision making [49]); total number of correct words from the Verbal Fluency Test (FAS), a phonemic test assessing the individual´s ability to generate words starting with the same letter [50]; and the Controlled Oral Word Association Test (COWAT), a semantic test assessing the individual´s ability to generate words belonging to the same semantic category [51] (see Table 1). 

Neuropsychological evaluations were performed by psychologists trained in the use of these instruments. Interrater reliability for administration and scoring of the cognitive scales were determined using the intraclass correlation coefficient in an independent sample of 10 subjects prior to the baseline assessments (>0.85 for all instruments). For this study purpose, raw scores were preferred over age-scale standardized scores because the later have small variances.

### 2.5. Statistical Analysis

Data were analyzed on an intention-to-treat basis. Normal distribution of variables was assessed by means of the Shapiro–Wilk test. Means and standard deviations (SDs) were used to describe continuous variables. Frequencies were used to describe discrete variables. Differences in demographic, neuropsychological, and clinical variables between participants in the PE and the NS group interventions at baseline were assessed by means of Student´s *t*-tests or chi-squared tests, according to the type of variable.

Spearman rank correlation and partial correlation analyses (adjusting for baseline cognitive performance, when appropriate) were used to examine the associations between changes (two-year follow-up minus baseline) in executive function tests and the variables and changes in the outcomes of clinical and functional measures within each treatment group. 

All variables were tested for collinearity assumptions. No autocorrelation or collinearity was observed in the a priori specified independent variables. Thus, corrections for multiple comparisons were not done because all comparison analyses were independent, they were specified a priori, and collinearity assumptions were met [52].

All statistical analyses were performed with SPSS version 18 (SPSS Inc., Chicago, Illinois, USA), and a two-tailed *p* value ≤0.05 was considered statistically significant.

## 3. Results

### 3.1. Sample Characteristics 

A sample of 22 adolescents with psychosis (mean age 16.32 ± 1.25; range (14–19)) were allocated to the experimental (PE; *N* = 10) and the control group (NS; *N* = 12), respectively, and completed clinical, functional, and neuropsychological assessments over time. We found no statistically significant differences between participants in the PE and the NS groups in sociodemographic, clinical, functional, antipsychotic treatment, or neuropsychological characteristics at baseline, with the exception of cognitive performance in the WCST number of categories completed which was higher in the NS group (t = −2,5, *p* = 0.019), the WCST number of errors which was higher in the PE group (t = 2.05, p = 0.053), and the COWAT number of correct words which was higher in the PE group (t = 2.19, *p* = 0.041) (see Table 2). Participants in both PE and the NS groups improved in clinical and functional outcome scores over time. In addition, there were no differences in the dose of antipsychotic treatment between the PE and the NS groups at baseline (t = 1.69, *p* = 0.11) or at two-year follow-up (t = 1.25, *p* = 0.43).

### 3.2. Associations between Change in Executive Function and Change in Functional and Clinical Variables at Two-Year Follow-Up in the PE and the NS Groups Separately

Because of the observed differences in cognitive performance at baseline between participants in the PE and the NS groups (i.e., specifically in the WCST number of categories completed, WCST number of errors, and the COWAT number of correct words), an initial Spearman’s rank-order and secondary partial correlations (adjusting for baseline cognitive performance scores as appropriate) were conducted between changes (two-year follow-up minus baseline) in executive function and change in clinical and functional variables at the two-year follow-up (see Table 3 and Table 4).

When examining initial Spearman’s rank-order correlation, significant, negative correlations were found between increased verbal fluency (i.e., change in FAS number of correct words) (r = −0.73, *p* = 0.025, 95% CI −0.932, −0.192) and change (decrease) in PANSS negative symptoms in the PE group. In addition, increased verbal semantic fluency (i.e., change in COWAT verbal fluency categories) (r=−0.75, *p* = 0.020, 95% CI −0.936, −0.224) was significantly correlated with change (decrease) in PANSS positive symptoms in this PE group (see Table 3). No significant associations were found for executive function performance measures and global functioning (GAF), the number of hospital visits, nor the number of hospital days.

When we controlled for baseline performance scores in COWAT verbal fluency categories on the relationship between change in COWAT verbal fluency categories and clinical and outcome measures, we found that improved positive and negative symptoms over time, assessed using the PANSS positive and PANSS negative change scores, were significantly associated with a change (increase) of COWAT verbal fluency categories (partial correlations: PANSS positive r = −0.76, *p* = 0.030, 95% CI −0.936, −0.224; PANSS negative r = −0.76, *p* = 0.029, 95% CI −0.907, −0.03) in the PE group. This indicated that baseline performance scores in COWAT had an influence on controlling for the relationship between change in PANSS and COWAT verbal fluency scores (Table 3). 

Significant partial correlations were also found between change (reduction) in the number of emergency department visits and increased semantic verbal fluency (COWAT; r = −0.74, *p* = 0.035, 95% CI −0.0908, −0.037) in the PE group (see Table 3). No associations were found for any of the executive function measures and clinical variables in the NS group (see Table 4).

## 4. Discussion

The present study suggests an association between an improvement in executive functioning performance and a reduction in clinical symptoms and the number of visits to the emergency department in adolescents with psychosis who participated in a problem solving based PE group intervention. In contrast, these associations were not observed for adolescents in the NS group. Our results suggest that cognitive/executive functioning performance may play a role in clinical and daily life improvement in participants in the PE group, with increased semantic and phonetic verbal fluency abilities contributing to the specific symptoms (i.e., positive and negative) and reduced number of visits to the emergency department over time in this particular group. These findings indicate that implementation of problem-solving strategies within psychosocial PE interventions may have an impact on clinical and functional outcomes, by providing patients with long-lasting resources to manage daily life more effectively through enhancement of their executive functioning abilities. 

In the current study, we found particular associations between increased verbal fluency abilities and decreased positive and negative symptoms and reduced number of visits to the emergency department in a group of adolescents with psychosis attending a problem solving based PE group intervention. Specific linguistic abilities such as verbal fluency are crucial cognitive processes that have been implicated as contributing to global impairments in executive function [50]. The abilities involve more than semantic or phonetic knowledge, requiring participants to access their mental lexicon and semantics to retrieve words. They also involve executive skills to track prior responses, select words meeting certain constraints, and block intrusions from other semantic categories [54]. Thus, deficits in verbal/language ability and executive control would cause poor performance in the verbal fluency tasks. In this regard, the retrieval process in verbal fluency tasks such as FAS or COWAT is considered a measure of executive function, as it requires planning, monitoring, and decision making in order to inhibit irrelevant information and select the correct responses (i.e., a limited set of words from lexical/phonetic or semantic memory) [9,50,55]. As such, these tasks (i.e., phonetic and semantic) have been included in the present study as a measure of executive function, providing an indicator of distinct cognitive mechanisms that have shown to have an effect on reducing symptoms and the number of emergency visits, which might be reflective of improved symptoms and crisis management of subjects in the PE group through enhancement of their executive skills/verbal fluency abilities, which has not been observed in participants in the NS group. 

Previous studies on the relationship between cognitive function and clinical outcomes have outlined associations between verbal fluency deficits and the severity of negative symptoms [56]. In addition, improvements in verbal memory and executive functions with median to large effect sizes have been observed in cognitive remediation approaches for adolescent psychosis together with improvements in daily living, adaptive functioning, and family burden [57]. Furthermore, particular improvements in verbal fluency measures, together with significant positive and negative symptom reductions, have also been observed in specifically targeted problem-solving program interventions [58]. Verbal fluency performance has shown to be one of the most impaired cognitive functions in first-episode schizophrenia patients and individuals at ultra-high-risk for psychosis (i.e., specifically semantic verbal fluency [59] and one of the best cognitive predictors for psychosocial functioning [60]). The executive function verbal fluency tasks can also be used as an instrument for general verbal/language functioning. Thus, an improved ability of the subjects to express themselves and/or to actively engage in a conversation may result both in symptom and functional improvements. Studies have demonstrated that patients who have better communication skills are more autonomous and have better capacities to solve daily problems [61]. In our current study, improved verbal executive abilities might reduce severity of symptoms and the number of contacts with the emergency services as a result of the implementation of our problem solving based PE intervention. As we have previously hypothesized [2], our current data demonstrate that reductions in symptoms and emergency visits are associated with improved verbal/executive abilities within our problem-solving PE group, which is not present in individuals attending the NS group intervention. It may also be hypothesized that symptom reduction, per se, has resulted in an improvement in the patients´ executive functioning performance. However, we have observed positive and negative symptom reductions in both PE and NS groups, with the reported associations between executive functioning performance and symptom reduction observed only in the PE group. These findings are congruent with the sort of problem-solving strategies that have been implemented within our PE intervention, where the participants need to generate the largest number of alternative solutions possible and make use of their verbal/executive processing abilities. In fact, even though not initially designed to address this matter, it is this particular cognitive (executive function) component that differentiates our problem-solving PE intervention from the NS program [1].

Limitations of this study include the small sample size, which may lead to unrepresentative treatment groups in comparison to the main study from which this sample was derived. Aligned with its main aims, the primary outcomes of the PIENSA clinical trial were defined as symptom and functional changes over time, not cognition. Hence, cognitive functioning assessments were not completed by all participants. On that account, the inclusion of a problem-solving component in the PE group was aimed at preventing relapses and facilitating self-management in stressful situations not specifically targeted to enhance remediation of executive function, which restricts our findings. A specially designed PE intervention that specifically addresses the executive/cognitive component is necessary for a better understanding of the reported associations and the unravelling of its pathway. Even though we aimed to control for plausible confounding factors, we found differences in cognitive performance at baseline between individuals in the PE and in the NS groups in our sample. Improvement in executive functioning performance may not be the only therapeutic component contributing to the observed beneficial effects of the PE intervention. Future studies should evaluate individual components of the PE intervention that are missing from the NS group, such as better medication adherence, which may affect the improvement in symptoms and functioning in the PE group. In this regard, an additional limitation is the lack of an objective measure of adherence to the antipsychotic treatment.

Despite these limitations, the present study suggests an avenue for further exploration in a field where there is still a lack of effective treatments. Negative and cognitive symptoms are prominent and persistent dimensions of psychosis that are not adequately treated with currently available psycho/pharmacotherapies, which have mainly targeted positive symptoms. Our results provide support for the inclusion of a cognitive component in traditional pharmaco/psychotherapies to enhance and extend its beneficial effects. Further, our results are consistent with the primary outcomes of the main PIENSA clinical trial, where PE intervention improved measures of negative symptoms and the number of visits to the emergency department in adolescents with psychosis at the end of the intervention [2] that persisted two years later [3]. The direction of this relationship between clinical and cognitive change still remains to be clarified. 

## 5. Conclusions

Our results highlight the PIENSA PE program may represent a significant advancement in psychosocial interventions for adolescent psychosis, as it uses a holistic approach that incorporates a cognitive remediation (i.e., problem-solving training) component that extends beyond traditional psychoeducation to facilitate the achievement of a broader symptom and functional recovery. This may have important implications in the development of new areas of clinical intervention focusing on the role of cognitive functioning in response to psychosocial treatments in psychosis.

## Figures and Tables

**Table 1:** Neuropsychological test and variables used to evaluate executive functioning performance at baseline and at two years of follow up.

Test	Executive Functioning Variable	Executive Ability
Trail Making Test (TMT)	TMT-A	Processing speed, sustained attention
	TMT-B	Cognitive flexibility, alternating attention, processing speed
	TMT Derived Score [TMTB-A]	Executive control
Verbal Fluency Test (FAS)	Total number of correct words	Verbal fluency
Verbal Fluency Categories Test (COWAT)	Total number of correct words	Verbal semantic fluency
Wisconsin Card Sorting Test (WCST)	Total number of perseverative errors	Cognitive flexibility
	Number of total errors	Executive control,
	Number of categories completed	planning, monitoring, and decision making

**Table 2:** Sociodemographic, clinical, and neuropsychological characteristics at baseline (*N* = 22).

	PE Group (*N* = 10)	NS Group (*N* = 12)	Total (*N* = 22)	Test PE vs. NS
Age, mean (SD)	16.40 (0.84)	16.25 (1.55)	16.32 (1.25)	t = 0.27, *p* = 0.79
Sex, male *N* (%)/female *N* (%)	4 (40%)/6 (60%)	9 (75%)/3 (25%)	13 (59%)/9 (41%)	χ^2^ =2.76, *p* = 0.19
Education, mean (SD)	10.30 (1.83)	8.83 (3.01)	9.50 (2.60)	t = 1.35, *p* = 0.19
Diagnosis, N (%) SSD/affective psychosis/other psychosis	4(40%)/2 (20%)/4 (40%)	5 (642%)/4(33%)/3 (25%)	9 (50%)/6 (27%) / 7(32%)	χ^2^ =0.275, *p* = 0.69
Ethnicity, N (%) Caucasian/other	9 (90%)/1 (10%)	9 (75%)/3 (25%) /	18 (81%)/4 (19%)	χ^2^ = 1.16, *p* = 0.56
Executive function measures, mean (SD)
Derived TMT score (TMTB-A)	60.60 (60.42)	83.67 (50.46)	73.18 (59.62)	t = −0.90, *p* = 0.38
FAS (total number of correct words)	31.22 (9.60)	29.58 (11.08)	30.29 (10.25)	t = 0.35, *p* = 0.73
COWAT (verbal fluency categories)	18.22 (1.57)	14.08 (1.14)	7.47 (21.95)	t = 2.19,***p* = 0.04**
WCST (number of perseverative errors)	22.30 (7.47)	18.75 (10.94)	20.36 (9.8)	t = 0.87, *p* = 0.40
WCST (number of total errors)	48.00 (15.30)	31.75(5.99)	39.14(19.85)	t = 2.05, ***p* = 0.05**
WCST (number of completed categories)	3.40 (1.78)	5.10 (1.32)	4.32 (1.73)	t = −2.5, ***p* = 0.02**
Clinical and functional outcome variables, mean (SD)				
PANSS positive	16.10 (9.157)	21.73 (10.23)	19.05 (10.09)	t = −1.30, *p* = 0.21
PANSS negative	18.60 (6.80)	20.45 (8.02)	19.57 (7.34)	t = −0.257 *p* = 0.58
GAF	67.89 (18.84)	52.58 (21.42)	59.14 (21.32)	t = 1.70, *p* = 0.11
Number of hospital visits	1.10 (0.32)	1.08 (0.29)	1.09 (0.29)	t = 0.129, *p* = 0.90
Number of hospital days	29.00 (12.53)	27.75 (8.81)	28.32 (10.41)	t = 0.274, *p* = 0.79
Number of emergency department visits	1.00 (0.67)	0.67 (0.49)	0.82 (0.59)	t = 1.234, *p* = 0.19
Antipsychotic treatment (chlorpromazine equivalents), mean (SD)	415.68 (118.21)	328.59 (247.54)	368.17 (200.14)	t = 1.69, *p* = 0.11

Differences between psychoeducational (PE) and nonstructured (NS) individuals at baseline based on a two-sample *t*-test (equal variance) and Pearson’s chi-squared tests. Significance was set at *p* ≤ 0.05. SSD, schizophrenia spectrum psychosis; CPZ, *Chlorpromazine (Chlorpromazine equivalents were used to derive the antipsychotic dosage at baseline and follow-up [53]); TMT, Trail Making Test; PANSS, Positive and Negative Syndrome Scale; GAF, Global Assessment of Functioning, Verbal Fluency Categories; and WCST, Wisconsin Card Sorting Test. Significant differences are marked in bold.

**Table 3:** Associations between change in executive functioning measures and change in clinical variables at two-year follow-up in the experimental PE group.

	PANSS Positive *rs/r [CI**]	*p*	PANSS Negative *r_s/_r[CI**]	*p*	GAF *r_s/_r [CI**]	*p*	Number of Hospital Visits *r_s/_r[CI**]	*p*	Number of Hospital Days *r_s/_r[CI**]	*p*	Number of Visits to the Emergency Department *r_s/_r [CI**]	*p*
**Derived TMT score [TMTB-A]**	0.092 [−0.57, 0.68]	0.814	0.192 [−0.497, 0.733]	0.620	0.118 [−0.552, 0.695]	0.763	−0.092 [−0.682, 0.57]	0.815	0.118 [−0.552, 0.695]	0.763	0.032 [−0.609, 0.648]	0.934
**FAS (total number of correct words)**	−0.0250 [−0.759, 0.45]	0.516	**−0.0733** [−0.932, −0.192]	**0.025**	0.209 [−0.484, 0.741]	0.589	0.183 [−0.504, 0.728]	0.638	−0.393 [−0.819, 0.314]	0.295	−0.387 [−0.817, 0.32]	0.303
**COWAT (verbal fluency categories)**	**−0.748** **[−0.936, −0.224]**	**0.020**	−0.647 **[−0.907, −0.03]**	0.060	0.557 [−0.111, 0.878]	0.119	−0.184 [−0.729, 0.504]	0.635	−0.186 [−0.73, 0.502]	0.632	−0.651 **[−0.908, 0.037]**	0.058
**COWAT (verbal fluency categories)** **†**	**−0.756** **[−0.988, −0.204]**	**0.030**	**−0.758** **[−0.984, −0.303]**	**0.029**	0.371[−0.367, 0.966]	0.366	−0.073 [−0.832, 0.697]	0.864	−0.217 [−0.949, 0.869]	0.605	**−0.743** **[−0.976, −0.077]**	**0.035**
**WCST (number of perseverative errors)**	−0.167 [−0.72, 0.52]	0.667	0.310 [−0.397, 0.786]	0.417	0.193 [−0.497, 0.733]	0.618	0.092 [−0.57, 0.682]	0.815	−0.034 [−0.649, 0.608]	0.932	0.292 [−0.413, 0.778]	0.446
**WCST (number of total errors)**	−0.452 [−0.84, 0.248]	0.222	−0.243 [−0.756, 0.456]	0.529	0.534 [−0.144, 0.87]	0.139	0.183 [−0.504, 0.728]	0.637	0.063 [−0.59, 0.666]	0.872	0.000 [−0.629, 0.629]	1.000
**WCST (number of Total errors) #**	−0.501 [−0.929, 0.183]	0.206	−0.139 [−0.933, 0.932]	0.743	0.392 [−0.515, 0.952]	0.337	0.254 [−0.861, 0.958]	0.543	0.256 [−0.589, 0.890]	0.541	0.129 [−0.789, 0.915]	0.760
**WCST (number of completed categories)**	0.147 [−0.531, 0.71]	0.705	0.078 [−0.58, 0.674]	0.842	−0.461[−0.845, 0.237]	0.212	0.285 [−0.419, 0.775]	0.458	−0.191 [−0.732, 0.498]	0.622	0.537 [−0.139, 0.871]	0.136
**WCST (number of completed categories) $**	0.456 [−0.555, 0.978]	0.257	−0.050 [−0.743, 0.666]	0.906	−0.292 [−0.916, 0.712]	0.483	0.079 [−0.855, 0.933]	0.852	−0.260 [−0.913, 0.883]	0.534	0.595 [−0.397, 0.962]	0.119

* rs = Spearman’s rank-order correlation coefficient. Significance set at *p* ≤ 0.05; ** 95% confidence interval; †, Partial correlation coefficient controlling for baseline COWAT (Verbal Fluency Categories) scores; ·#, Partial correlation coefficient controlling for baseline WCST (number of total errors) scores; $, Partial correlation coefficient controlling for baseline WCST (number of completed categories) scores; TMT, Trail Making Test; PANSS, Positive And Negative Syndrome Scale; FAS, FAS Verbal Fluency Task; GAF, Global Assessment of Functioning; COWAT, Verbal Fluency Categories; and WCST, Wisconsin Card Sorting Test. Significant differences are marked in bold.

**Table 4:** Associations between change in executive functioning measures and change in clinical variables at two-year follow-up in the control NS group.

	PANSS Positive *r_s/_r[CI**]	*p*	PANSS Negative *r_s/_r[CI**]	*p*	GAF *r_s/_r[CI**]	*p*	Number of Hospital Visits *r_s/_r[CI**]	*p*	Number of Hospital Days *r_s/_r[CI**]	*p*	Number of Visits to the Emergency Department *r_s/_r[CI**]	*p*
**Derived TMT score [TMTB-A]**	0.165 [−0.451, 0.674]	0.627	0.382 [−0.245, 0.784]	0.247	−0.361 [−0.774, 0.268]	0.248	−0.393 [−0.788, 0.233]	0.206	−0.315 [−0.752, 0.316]	0.318	−0.514 [−0.84, 0.084]	0.087
**FAS (total number of correct words)**	0.286 [−0.344, 0.738]	0.394	0.110 [−0.495, 0.643]	0.748	−0.071 [−0.619, 0.524]	0.828	−0.088 [−0.63, 0.511]	0.786	0.070 [−0.524, 0.619]	0.828	0.246 [−0.381, 0.718]	0.441
**COWAT (verbal fluency categories)**	−0.069 [−0.618, 0.525]	0.840	−0.339 [−0.764, 0.291]	0.308	0.213 [−0.411, 0.701]	0.505	0.263 [−0.366, 0.727]	0.408	0.150 [−0.463, 0.666]	0.643	0.345 [−0.285, 0.767]	0.273
**COWAT (verbal fluency categories) †**	0.016 0.582, 0.607]	0.965	−0.501 [−0.894, 0.300]	0.140	0.239 [−0.472, 0.626]	0.506	0.064 [−0.124, 0.649]	0.861	0.082 [−0.731, 0.656]	0.822	−0.108 [−0.740, 0.801]	0.766
**WCST (number of perseverative errors)**	−0.250 [−0.72, 0.378]	0.458	−0.046 [−0.603, 0.542]	0.893	0.078 [−0.519, 0.623]	0.810	0.484 [−0.124, 0.827]	0.111	0.067 [−0.527, 0.617]	0.836	0.074 [−0.522, 0.621]	0.819
**WCST (number of total errors)**	−0.073 [−0.62, 0.522]	0.830	−0.027 [−0.591, 0.555]	0.937	0.119[−0.488, 0.648]	0.712	0.306[−0.324, 0.748]	0.334	0.284[−0.346, 0.737]	0.372	−0.220[−0.704, 0.405]	0.491
**#**	−0.007 [−0.724, 0.650]	0.985	0.010[−0.533, 0.589]	0.978	0.035 [−0.654, 0.673]	0.924	0.205 [−0.025, 0.865]	0.570	−0.075 [−0.648, 0.824]	0.837	−0.587 [−0.920, −0.198]	0.074
**WCST (number of completed categories)**	0.482 [−0.127, 0.827]	0.134	0.268 [−0.361,.729]	0.425	−0.510[−0.838, 0.09]	0.090	−0.390 [−0.787, 0.236]	0.210	−0.468 [−0.821, 0.144]	0.125	0.192 [−0.429, 0.689]	0.551
**WCST (number of completed categories) $**	0.127 [−0.478, 0.728]	0.727	0.038 [−0.518, 0.739]	0.917	−0.155 [−0.879, 0.531]	0.670	−0.199 [−0.948, 0.000]	0.581	0.376 [−0.617,0.831]	0.284	0.419 [0.000, 0.766]	0.229

* rs = Spearman’s rank−order correlation coefficient. ** 95% confidence interval. Significance set at *p* ≤ 0.05; †, Partial correlation coefficient controlling for baseline COWAT (verbal fluency categories) scores; #, Partial correlation coefficient controlling for baseline WCST (number of total errors) scores; $, Partial correlation coefficient controlling for baseline WCST (number of completed categories) scores; TMT, Trail Making Test; PANSS, Positive And Negative Syndrome Scale; FAS, Verbal Fluency Task; GAF, Global Assessment of Functioning; COWAT, Verbal Fluency Categories; and WCST, Wisconsin Card Sorting Test.
